# Calorie restriction and cancer prevention: a mechanistic perspective

**DOI:** 10.1186/2049-3002-1-10

**Published:** 2013-03-07

**Authors:** Stephen D Hursting, Sarah M Dunlap, Nikki A Ford, Marcie J Hursting, Laura M Lashinger

**Affiliations:** 1 Department of Nutritional Sciences, The University of Texas at Austin, 1400 Barbara Jordan Blvd, DPRI 2.834, Austin, TX, 78723, USA; 2 Department of Molecular Carcinogenesis, The University of Texas-MD Anderson Cancer Center, Smithville, TX, USA; 3 Clinical Science Consulting, Austin, TX, USA

**Keywords:** Angiogenesis, Calorie restriction, Cancer prevention, Growth factors, Inflammation, Metabolism, Obesity

## Abstract

Calorie restriction (CR) is one of the most potent broadly acting dietary interventions for inducing weight loss and for inhibiting cancer in experimental models. Translation of the mechanistic lessons learned from research on CR to cancer prevention strategies in human beings is important given the high prevalence of excess energy intake, obesity, and metabolic syndrome in many parts of the world and the established links between obesity-associated metabolic perturbations and increased risk or progression of many types of cancer. This review synthesizes findings on the biological mechanisms underlying many of the anticancer effects of CR, with emphasis on the impact of CR on growth factor signaling pathways, inflammation, cellular and systemic energy homeostasis pathways, vascular perturbations, and the tumor microenvironment. These CR-responsive pathways and processes represent targets for translating CR research into effective cancer prevention strategies in human beings.

## Introduction

Calorie restriction (CR), a dietary regimen in which subjects (typically test animals) receive a reduced energy diet (typically, a 20 to 40% reduction in total energy intake relative to an unrestricted comparison group), is one of the most potent and broadly acting dietary interventions for preventing or reversing weight gain and inhibiting cancer in experimental tumor models [1]. Recent reports of decreased risk of diabetes, neurological degeneration, and cancer in response to CR in rhesus monkeys [2,3], and observations that CR decreases inflammatory and endocrine markers associated with increased breast cancer risk in women [4,5], suggest that the beneficial effects of CR on metabolism and chronic disease risk observed in rodent models may extend to human beings.

Observational epidemiologic studies provide additional evidence that CR exerts beneficial effects on longevity and cancer risk in human beings [1]. For example, inhabitants of Okinawa, Japan, who until recently had consumed significantly fewer calories than residents of the main Japanese islands, have always had lower death rates from cancer and other chronic diseases than inhabitants of the Japanese mainland [6]. In addition, patients with early-onset anorexia nervosa, and hence periods of energy restriction, have reduced risk of breast cancer [7]. Furthermore, surveillance data from some populations exposed to varying degrees of energy restriction during World War II are also consistent with the hypothesis that CR decreases cancer risk. For example, Norwegian women showed reduced breast cancer risk later in life in association with acute (<1 year) energy restriction (≈50% reduction in calorie intake without significant changes in diet quality) [8]. However, the confounding effects of severe physical and psychosocial stress, malnutrition, infection, and other factors associated with war conditions make many of these studies a challenge to interpret. Populations with more severe restriction than experienced in Norway, such as survivors of the 1944 Dutch ‘Hunger Winter’, the Jewish Holocaust, and the Siege of Leningrad, actually displayed higher breast cancer rates [9-11], indicating a threshold beyond which undernutrition (especially when combined with other stressors) might be cancer promoting. This is particularly true for those born around the time of the severe deprivation and stress, suggesting an important perinatal window of susceptibility to metabolic reprogramming [12].

These stressful conditions are in contrast with the controlled conditions characteristic of most CR studies in animal models that consistently show anticancer effects. Calorie restriction regimens are often referred to as ‘CR with optimal nutrition’ or ‘undernutrition without malnutrition,’ and CR experiments typically involve 20 to 40% reductions in total energy relative to *ad libitum*-fed controls, but with adequate nutrition and a controlled physical environment [1]. In rodent models, CR regimens administered throughout life are generally more effective against cancer than CR regimens initiated in adulthood, although both early-onset and adult-onset CR, relative to control diet regimens, are protective against a variety of cancer types [1]. In the two published rhesus monkey studies to date, there was a consistent anticancer effect of CR when begun in young adults [2,3]. However, in the latest study by Mattison and colleagues [3], there was no anticancer effect of CR when begun in older adults, and there was no effect of CR, regardless of age of onset, on overall survival. This is in contrast with the earlier report by Weindruch and colleagues [2] showing both anti-aging and anticancer effects of CR. Several differences between the studies may account for their differential findings. The Weindruch group, relative to the Mattison group, used a more purified, energy-dense diet that was ≈30% sucrose (versus 4% sucrose in the Mattison study). Thus the Weindruch group’s controls, relative to the Mattison group’s controls, were more obese and less healthy, and hence their CR monkeys had a greater difference in weight and metabolic parameters. The diets fed to the monkeys in the Mattison study also contained fish oil, which probably contributed further to their monkeys being healthier and more metabolically similar regardless of caloric intake. Differences in genetics may also have contributed to the observed differences, as although both studies used rhesus monkeys, the monkeys originated from different countries. Nonetheless, there is evidence that CR can prevent cancer in monkeys, with the magnitude of the effect dependent on several factors, including age of onset, nutritional quality of the diet, and genetic susceptibility.

Several clinical trials funded by the National Institute of Aging are currently being conducted to address the question of whether the observed health benefits of CR in rodents and nonhuman primates translate to human beings. One of these trials, the Comprehensive Assessment of Long-Term Effects of Reducing Intake of Energy (CALERIE) Study, is evaluating the effects of a 2-year CR regimen (25% less energy than controls) in healthy, nonobese individuals. Preliminary reports on CALERIE indicate that many of the same metabolic and endocrine changes observed in rodents and monkeys are also occurring in human beings in response to CR [13,14]. These findings are consistent with recent studies in women at high risk for breast cancer showing that inflammatory and growth factor signaling pathways are reduced by total CR or 2 days/week of restricted carbohydrate calories [4,5]. The observed metabolic effects of 2-days/week of restricted carbohydrate calories are of particular interest, since it is probably easier and more sustainable for most people to restrict a single macronutrient, such as carbohydrates, periodically than to restrict total energy chronically.

**Figure 1:**
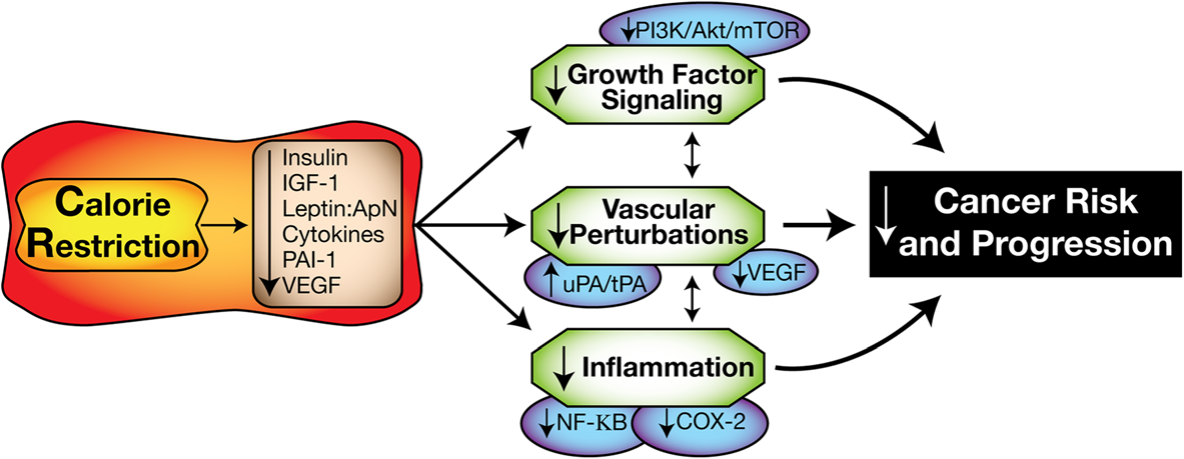
**Calorie restriction and cancer: overview of mechanisms.** Chronic exposure to a calorie restriction regimen results in reduced circulating levels of several hormones, growth factors and cytokines, leading to decreased growth factor signaling, fewer vascular perturbations, and decreased inflammation. Together, these responses to calorie restriction result in decreased cancer risk and progression. An arrow preceding text denotes a directional effect (eg, activity or concentration). Abbreviations: IGF-1, insulin-like growth factor-1; ApN, adiponectin; PAI–1, plasminogen activator inhibitor–1; tPA, tissue-type plasminogen activator; uPA, urokinase-type plasminogen activator; VEGF, vascular endothelial growth factor; PI3K, phosphoinositide 3-kinase; mTOR, mammalian targt of rapamycin; NF-kB, nuclear factor kB; COX-2, cyclooxygenase-2.

In this review, we discuss possible mechanisms underlying the anticancer effects of CR, with emphasis on CR-associated changes in growth signaling, inflammation, and angiogenesis, as well as emerging evidence suggesting that autophagy and the sirtuin pathway may also play roles in the effects of CR on tumor development and progression. As summarized in Figure 1, we specifically describe the dysregulation of growth signals (including insulin, IGF-1, adipokines, and their downstream signaling pathways), inflammatory cytokines and cellular crosstalk, and vascular integrity factors, in response to CR, and suggest that these multifactorial CR-induced changes combine to suppress tumor development or progression. Components of these interrelated pathways offer possible mechanism-based targets for the prevention and control of cancers, particularly the estimated 20% [15] of human cancers related to, or caused by, excess body weight and the metabolic syndrome.

## Calorie restriction impacts growth signals

### Insulin, insulin-like growth factor (IGF)-1, and glucose

The peptide hormone insulin is produced by beta cells in the pancreas and released in response to hyperglycemia, which is associated with insulin resistance, aberrant glucose metabolism, chronic inflammation, and the production of other metabolic hormones, such as IGF-1, leptin, and adiponectin [16]. Clinical and epidemiologic evidence suggests that elevated levels of circulating insulin or the cleavage product of proinsulin (C-peptide) are associated with increased risk or progression of cancers of the breast (pre- and postmenopausal), endometrium, colon, kidney, and pancreas [16,17]. High circulating levels of insulin also upregulate hepatic synthesis of IGF-1 critical to growth and development of many tissues, particularly during the prenatal period [16,18]. In the circulation, IGF-1 is typically bound to IGF-binding proteins (IGFBPs) that regulate the amount of free IGF-1 bioavailable to elicit growth or survival signaling [16,18]. An elevated level of circulating IGF-1 is an established risk factor for many cancer types [16-20].

The decrease in insulin and IGF-1 levels in response to CR is due, at least in part, to reduced glucose levels [18]. In the hyperinsulinemic state (as commonly occurs with obesity), higher insulin levels in the portal circulation in response to hyperglycemia upregulate the growth hormone receptor (GHR) and augment GHR signaling, increasing hepatic IGF-1 production. Insulin resistance and hyperinsulinemia are also associated with downregulation of IGFBPs, increasing the levels of bioavailable IGF-1. In contrast, the enhanced insulin sensitivity and normalized glucose levels in response to a CR regimen, relative to a control or diet-induced obesity (DIO) regimen, results in lowered serum insulin and IGF-1, and increased IGFBP production, particularly IGFBP1 and 3 (and hence low levels of bioavailable IGF-1). The CR-induced reduction in glucose may also have direct anticancer effects. In cancer cells, mitochondrial metabolism of glucose is reprogrammed to meet the demands of macromolecular synthesis required for cellular proliferation. This metabolic switch of glucose metabolism from oxidative phosphorylation to oxidative glycolysis (first described by Otto Warburg in 1924) is now understood to be necessary to supply sufficient nucleotides, lipids, and proteins for daughter cell production [21]. Cancer cells do this, however, at the expense of substrate inflexibility relative to normal cells, as the increased proliferation rate associated with most cancer cells can only be sustained by a constant supply of the necessary building blocks derived from the flux of glucose carbons through glycolysis. Thus, it is possible that precancerous or cancer cells undergoing this metabolic reprogram, and hence developing a glucose addiction, may have heightened sensitivity to reductions in glucose levels, as occurs with CR.

Insulin signals through the insulin receptor (IR), of which there are two isoforms, IR-A and IR-B, formed by the absence or presence of exon 11, respectively [16,18]. IR-A expression has been demonstrated in fetal cells and many tumor cells, and signaling through IR-A results in more mitogenic effects than IR-B signaling, which activates the metabolic signaling pathway. Hyperinsulinemia, therefore, can activate signaling pathways that lead to both metabolic and mitogenic effects. IGF-1 primarily signals through the IGF-IR, and mediates mitogenic effects. Cells that express the IR and IGF-IR can also express hybrid receptors, made of the α and β subunit of an IR (IR-A or IR-B) bound to the α and β subunit of the IGF-IR. Insulin has negligible affinity for either configuration, while IGF-1 can bind efficiently to either of these hybrid receptors. Increased expression of IR-A in tumors therefore allows for the increased formation of IGF-IR/IR-A hybrid receptors in tumors, facilitating mitogenic signaling by IGF-1 through the hybrid receptor or insulin. Taken together, it is clear that either hyperinsulinemia or increased IGF-1 (or both) can augment tumor growth by signaling through these receptors.

The phosphatidylinositol-3 kinase (PI3K)/Akt pathway, downstream of both the insulin receptor and IGF-IR, is one of the most commonly activated pathways in epithelial cancers [21]. This pathway integrates intracellular and environmental cues, such as growth factor concentrations and nutrient availability, to regulate cellular survival, proliferation, protein translation, and metabolism. Akt regulates the mammalian target of rapamycin (mTOR) [22], which regulates cell growth, cell proliferation, and survival through downstream mediators. Increased activation of mTOR is common in tumors and many normal tissues from obese or diabetic mice, while CR decreases mTOR signaling in these same tumors and normal tissues [23]. Moreover, mTOR activation is inhibited by increased AMP-activated kinase (AMPK) under low nutrient conditions [24]. Specific mTOR inhibitors block the tumor-enhancing effects of obesity in mouse models [25,26].

### Adiponectin, leptin, and the leptin: adiponectin ratio

Adiponectin is a peptide hormone primarily secreted from visceral white adipose tissue. In contrast to leptin and other adipokines, circulating levels of adiponectin negatively correlate with adiposity, and are thus increased by CR and decreased by obesity [27]. Adiponectin functions to counter obesity-related metabolic perturbations, such as insulin resistance and leptin resistance, that impact glucose and fatty acid metabolism, alter insulin responses, and increase production of inflammatory cytokines [27]. Thus, possible mechanisms through which adiponectin exerts anticancer effects may include increasing insulin sensitivity and decreasing insulin/IGF-1 and mTOR signaling via activation of AMPK [28]. Adiponectin also reduces proinflammatory cytokine expression via inhibition of the nuclear factor κ-light-chain-enhancer of activated B-cells (NF-κB) [28,29].

Leptin is a peptide hormone produced by white adipose tissue, and the leptin receptor is a member of the class I cytokine receptor family that signals through the Janus kinase and signal transducer activator of transcription (JAK/STAT) pathway commonly dysregulated in inflammatory conditions and many cancers [30,31]. Circulating leptin levels positively correlate with adipose stores and nutritional status, and function as an energy sensor to signal the brain to reduce appetite. Leptin has direct effects on peripheral tissues, indirect effects on neuroendocrine regulators of appetite and energy expenditure in the hypothalamus, and impacts carcinogenesis, angiogenesis, immune responses, cytokine production, and other biological processes [31]. In the obese state, adipose tissue overproduces leptin, and the brain no longer responds to the signal, resulting in leptin resistance. Insulin, glucocorticoids, tumor necrosis factor-α (TNF-α), and estrogens all stimulate leptin release [31]. Calorie restriction consistently and robustly decreases systemic leptin levels in a manner dependent on the extent of the adiposity loss [1].

*In vitro*, animal, and epidemiologic evidence linking adiponectin [32-36] or leptin [37-39] individually to cancer risk is mixed. Intermittent CR suppresses murine mammary tumor incidence in association with decreased leptin-to-adiponectin ratio [32]. Associations between the leptin-to-adiponectin ratio and the metabolic syndrome [40-42] and some cancers [43-45] have been reported, but further characterization of these links is needed.

### Calorie restriction decreases chronic inflammation

Chronic inflammation is characterized by increased circulating free fatty acids, cytokines, and chemokines that attract immune cells (such as macrophages that also produce inflammatory mediators) into the local microenvironment [46-48]. The inflammatory cascade is further amplified by the release of inflammatory cytokines, such as interleukin (IL)-1β, IL-6, TNF-α, and monocyte chemoattractant protein-1, primarily from macrophages, into the local and systemic circulation. Adipocytes can enlarge past the point of effective oxygen diffusion, which results in hypoxia and eventually necrosis. Free fatty acids escape the engorged or necrotic adipocytes and deposit in other tissues, and this in turn promotes insulin resistance, diabetes (through downregulation of insulin receptors and glucose transporters), hepatic steatosis, and pancreatic steatosis, and also activates signaling molecules involved in epithelial carcinogenesis, such as NF-κB and cyclooxygenase (COX)-2 [49]. The transcription factor NF-κB is activated in response to bacterial and viral stimuli, growth factors, and inflammatory molecules (for example, TNF-α, IL-6, and IL-1β), and is responsible for inducing gene expression associated with cell proliferation, apoptosis, inflammation, metastasis, and angiogenesis. Activation of NF-κB is a common characteristic of many tumors and is associated with insulin resistance and elevated circulating levels of leptin, insulin, or IGF-1 [46,50,51].

A connection between chronic inflammation and cancer development was observed 150 years ago when Rudolph Virchow noted an abundance of leukocytes in neoplastic tissue [52]. Inflammation is now considered a hallmark of cancer, and evidence is accumulating that chronic, ‘smoldering’ inflammation is associated with increased cancer risk [53-55]. Indeed, several tissue-specific inflammatory lesions are established neoplastic precursors for invasive cancer, including inflammatory bowel disease for colon cancer, pancreatitis for pancreatic cancer, dermatitis for certain forms of skin cancer, and gastritis for gastric cancer [56,57]. Tumor and preneoplastic microenvironments are composed of mixtures of cell types including epithelial cells, fibroblasts, mast cells, and cells of the innate and adaptive immune system [58]. As discussed previously, macrophages, which are activated in the obese state, infiltrate tumors and amplify the inflammatory tumor microenvironment, often through NF-κB-dependent production of cytokines and angiogenic factors [58]. COX-2 is another important cancer-related inflammatory mediator that is upregulated in most tumors and catalyzes the synthesis of the potent inflammatory lipid metabolite, prostaglandin E_2_. COX-2 expression, an indicator of poor prognosis in many cancer types, is increased in response to obesity [59].

Calorie restriction can prevent much of the inflammation associated with preneoplasia or neoplasia [46,60-62]. Specifically, CR decreases the number of tumor-infiltrating macrophages, levels of circulating and tissue cytokines, and NF-κB signaling and COX-2 expression in many tissues and tumor types [46,61,62]. Thus evidence is accumulating that the anti-inflammatory effects of CR contribute significantly to its cancer preventive effects [1,46].

### Calorie restriction abrogates vascular perturbations

Imbalances in the production or interactions of several factors influence key functions of the endothelium, including its roles in regulating angiogenesis, hemostasis, vascular density, inflammation, and vascular wall integrity. One such factor is PAI-1, a serine protease inhibitor produced by endothelial cells, stromal cells, and adipocytes in visceral white adipose tissue [63]. PAI-1, through its inhibition of urokinase-type and tissue-type plasminogen activators, regulates fibrinolysis and integrity of the extracellular matrix [64]. Increased circulating PAI-1 levels, frequently found in obese subjects, are associated with increased risk of atherogenesis and cardiovascular disease, diabetes, and several cancers [63-66]. PAI-1 is also involved in angiogenesis and thus may contribute to obesity-driven tumor cell growth, invasion, and metastasis [66]. Circulating levels of PAI-1 are consistently decreased in response to CR [1], although the mechanistic link between PAI-1 and cancer requires further study.

Another important mediator of vascular integrity is the heparin-binding glycoprotein vascular endothelial growth factor (VEGF) produced by adipocytes and tumor cells. VEGF has mitogenic, angiogenic, and vascular permeability-enhancing activities specific for endothelial cells [67]. The need for nutrients and oxygen triggers tumor cells to produce VEGF, which leads to the formation of new blood vessels (angiogenesis) to nourish the rapidly growing tumor. VEGF may also facilitate the metastatic spread of tumors cells [68]. Adipocytes communicate with endothelial cells by producing a variety of pro-angiogenic and vascular permeability-enhancing factors, including VEGF and PAI-1 [69]. In the obese, nontumor setting, these factors stimulate neovascularization in support of the expanding fat mass [69]. Circulating levels of VEGF are increased in obese, relative to lean, human beings and animals, and increased tumoral expression of VEGF is associated with poor prognosis in several obesity-related cancers [70-73]. Data to date for several experimental tumor models [71-73] suggest that CR decreases systemic and tissue VEGF and has anti-angiogenic effects.

## Emerging mechanisms underlying the anticancer effects of calorie restriction

### Sirtuins

The sirtuin family of proteins has been implicated in the regulation of endocrine signaling, stress-induced apoptosis, and the metabolic changes associated with energy balance modulation and aging [74-76]. Sirtuins were originally studied in yeast and nematodes, where CR increases lifespan in association with the levels and activity of the Sir2 protein [77-79]. The levels of Sir2, or its mammalian homolog SIRT1, rise in response to CR [75-79]. SIRT1 is an NAD-dependent deacetylase that inhibits stress-induced apoptotic cell death, and may modulate IGF-1, adiponectin, and insulin production, and insulin sensitivity, in different tissues [79-81].

The specific roles of sirtuins in cancer development or progression are not yet clear. SIRT1 is upregulated in several tumor types and can inhibit apoptosis and downregulate the expression of tumor suppressor genes to enhance survival of epithelial cancer cells [82-85]. In addition, the SIRT1 activator SRT1720 promotes tumor cell migration and lung metastases in a murine breast cancer model [86]. In contrast, there is also evidence that SIRT1 can act to suppress polyp formation in the APC^Min^ intestinal tumor model [87]. Additionally, in preclinical studies the phytochemical resveratrol activates SIRT1 and reduces cancer development in several models [88]. SIRT1-overexpression did not influence the anticancer effects of an every-other-day fasting regimen (a variation of CR) in a p53-deficient mouse model of cancer, suggesting that SIRT1 may have a limited role in the effects of CR on cancer [89]. Given the conflicting data to date regarding the tumor-enhancing, versus inhibitory, effects of SIRT1 activation, and the apparently limited role of SIRT1 in the response to CR, it remains unclear whether SIRT1 or other sirtuins represent mechanistic targets for cancer prevention.

### Autophagy

Autophagy is a cellular degradation pathway involved in the clearance of damaged or unnecessary proteins and organelles. It also provides an alternative source of energy and substrates during periods of restricted dietary intake (such as CR) or metabolic stress to enhance survival. In response to a 30% CR regimen (relative to an *ad libitum*-fed control diet), fasting plasma glucose levels and insulin secretion are reduced (and insulin sensitivity is increased), and glucagon is released from the alpha cells of the pancreas, resulting in increased autophagy in the liver, beta cells of the pancreas, and skeletal muscle [90,91]. One of the proposed mechanisms of CR is that under conditions of nutrient limitation, there is a shift in metabolic investment from cell replication and growth to maintenance, to ensure extended survival [92]. This tightly regulated process is driven by a group of autophagy-related proteins, and is suppressed by the conserved nutrient sensor TOR [93]. CR regulates TOR complex 1 and, to a lesser extent TOR complex 2, in many species, including flies, worms, yeast, and mammals. TOR complex 1 signaling regulates protein translation and many cellular processes, including metabolism and autophagy [93]. In addition, suppression of nutrient-activated TOR signaling is sufficient to trigger an energy stress response that is coordinated by AMPK, and this metabolic program blunts the growth responses to nutrient availability and promotes autophagy [94].

Several longevity-promoting regimens, including inhibition of TOR with rapamycin, resveratrol, or the natural polyamine spermidine, may require autophagy for their effects [95]. Autophagy activation is essential for clearing cellular damage and disease prevention in normal cells, and tumor cells also utilize autophagy to maintain a favorable metabolic state for daughter cell production, especially under limiting nutrient conditions [96]. However, little is known about what role autophagy plays in CR-mediated effects on tumor development or progression.

### Calorie restriction mimetics

The identification and development of natural or synthetic agents that mimic some of the protective effects of CR may facilitate new strategies for cancer prevention. Given how difficult it is for many people to adopt a low-calorie diet for an extended period, the identification of drugs or other agents that could either complement or even reproduce the anticancer effects of CR without drastic changes in diet and lifestyle is a goal for many pharmaceutical companies. Numerous studies have used microarray analyses to profile the molecular targets responding to CR and other dietary energy balance modulations [97-101]. Most of these studies were focused on understanding CR effects related to aging, and they revealed that the extent to which CR modulates the transcriptome is species-specific, tissue-specific, and dependent on the duration and intensity of CR. Nonetheless, some emerging patterns from these studies suggest that transcripts involved in inflammation, growth factor signaling (particularly related to the insulin and IGF-1 pathways), oxidative stress, and nutrient metabolism are commonly altered by CR. Application of the emerging field of metabolomics to this question should accelerate the identification of additional targets.

Genetic induction of the Sir2/SIRT1 family of NAD-dependent deacetylases mimics some of the effects of CR [75,77,78,87], although the role of SIRT1 in the anticancer effects of CR is unclear and may be minimal [89]. Sirtuin modulators, including resveratrol and its analogs, and pharmacologic modulators of SIRT1 [82], exert some anticancer activity, although much of this work has been limited to *in vitro* systems and awaits verification *in vivo*.

The IGF-1 and Akt/mTOR pathways, in addition to the sirtuin pathway, have emerged as potential key mediators of CR’s anticancer effects, and are the most promising initial targets for possible CR mimetics. Agents or interventions that safely reduce IGF-1, or inhibit one or more components of the signaling pathways downstream of IGF-1 and other growth factors (including Akt and mTOR) without requiring drastic dietary changes, may provide an effective physiological or pharmacological mimetic of those effects. The hope is that these agents or interventions could be readily adopted by a large proportion of the population, particularly those unable to lose weight and at high risk of cancer or other chronic diseases associated with obesity.

As recently reviewed [16,102], antireceptor antibodies, small-molecule receptor kinase inhibitors, and (to a lesser extent) anti-IGF ligand antibodies are being developed to target the IR or IGF-1 receptor, and several promising agents from each of these classes have advanced to clinical trials. The antireceptor antibodies have been the subject of the most intense translational research activity, extending to phase 3 trials, while the other classes are currently in phase 1 or phase 2 trials. The various antireceptor antibodies that have been developed were designed to avoid IR inhibition (blocking IR would be likely to have significant adverse effects), and this is generally being accomplished. Each targets ligand binding to the IGF-IR, and preliminary evidence suggests that the effects extend to hybrid receptors. Despite the lack of interference with insulin binding, the use of these antibodies causes hyperglycemia and hyperinsulinemia, and can also lead to increased levels of serum IGF-1 in compensation for the reduced IGF-IR signaling. This can contribute to insulin resistance in patients receiving these antibodies, and these untoward effects, along with generally disappointing trial results to date, is limiting the pace of development of these agents [102].

Although the initial development of small-molecule tyrosine kinase inhibitors involved attempts to achieve IGF-IR specificity, the newer agents tend to partially inhibit several members of the insulin and IGF-1 receptor family, which may limit side effects and provide a therapeutic advantage of more specific inhibitors. Early clinical experience suggests that these agents are safer than was originally anticipated, possibly because the drug concentrations that are achieved are fairly low in muscle, which is a major metabolic regulator, perhaps accounting for a modest rather than a severe effect of these kinase inhibitors on metabolic perturbations. Nevertheless, insulin levels are generally increased in patients treated with these kinase inhibitors, possibly limiting their efficacy and the pace of their development [16].

In addition to pharmacological agents targeting these receptors or ligands, including emerging work on microRNA-based approaches [103], a wide variety of natural agents with demonstrated cancer chemopreventive or chemotherapeutic activity have recently been reported to target components of the insulin/IGF-1 pathway [104]. These agents, which probably exert only modest inhibitory effects on insulin/IGF receptor activity, may provide a promising and safe approach, especially if effective combinations can be identified, to breaking the obesity-cancer link

Pharmacological mTOR inhibitors have emerged as lead candidates for CR mimetics. Rapamycin treatment extends lifespan and delays cancer in mice, providing additional support for mTOR as a target for mimicking the effects of CR [105]. We have shown that rapamycin or its analog, RAD001 (everolimus), can offset the obesity-associated increased growth of mammary or pancreatic tumors [25,62]. Rapamycin is a potent inhibitor of the mTOR complex 1, but chronic rapamycin exposure has been linked in some studies to disruption of mTOR complex 2 signaling, resulting in impaired glucose tolerance and insulin action [106]. Thus while inhibiting mTOR complex 1 appears to be a good strategy for mimicking many of the anticancer effects of CR, the search for agents that can do so without disrupting mTOR complex 2 signaling is ongoing.

An mTOR-inhibiting drug with great promise as a CR mimetic that overcomes the concerns about glucose intolerance associated with rapamycin is metformin, a biguanide commonly used to treat type 2 diabetes [107]. Metformin inhibits gluconeogenesis through indirect activation of AMPK in the liver and possibly cancer cells, and may also exert direct effects on AMPK in cancer cells [107]. Administration of metformin suppresses tumor development or growth in multiple experimental models, including colon, mammary, and hematopoietic cancer models [107]. Epidemiological studies have suggested that type 2 diabetic patients treated with metformin have a lower risk of developing from or dying from cancer, relative to diabetic patients receiving sulfonylurea, insulin, or other therapies [108-110]. A randomized trial is now underway to evaluate the effect of metformin on breast cancer recurrence [111]. Phenformin, another biguanide that has been abandoned for diabetes therapy due to its toxicity from lactic acidosis is a more potent AMPK inhibitor than metformin and may also have some potential as a CR mimetic at lower, nontoxic doses [107].

An emerging issue in the area of mTOR inhibitors as CR mimetics is that of the relative effects of nature versus nurture, that is, the contribution of systemic factors (which has been the focus of this review) in the context of cell autonomous effects. The observations of Kalaany and Sabatini [112] that cancer cells with constitutively activated PI3K mutations are proliferative *in vitro* in the absence of insulin or IGF-1 and form CR-resistant tumors *in vivo* illustrate this issue. We also found that constitutive activation of mTOR in MMTV-Wnt-1 mammary tumor cells blocked the anticancer effects of CR [26]. These findings suggest that cell autonomous alterations, such as activating mutations of PI3K or downstream mTOR pathway components, may influence the response of cells to CR or CR mimetics.

Another emerging issue is that, in addition to impacting the growth and survival of aberrant cells, CR and mTOR inhibition may also affect the stem cell compartment and enhance maintenance or repair of tissues. Yilmaz and colleagues [113] showed that CR, through its inhibitory effects of mTOR signaling in Paneth cells (immune-related support cells in the stem cell niche) adjacent to intestinal stem cells, preserves and even enriches intestinal stem cells. The augmenting effects of CR (via Paneth cells) on intestinal stem cell self-renewal can be mimicked by rapamycin. Cerletti *et al*. [114] similarly found that CR enriches skeletal-muscle stem cells and increases their regenerative potential. In addition, we showed that mammary tumors highly enriched in breast cancer stem cells have heightened sensitivity to the anticancer effects of CR [115]. Specifically M-Wnt cells, cloned from a spontaneous mammary tumor from a MMTV-Wnt-1 transgenic mouse, display a mesenchymal morphology, stably express stem cell markers, and rapidly generate claudin-low mammary tumors when orthotopically injected into syngeneic C57BL/6 mice. Calorie reduction almost completely ablates M-Wnt tumor growth relative to tumors induced by E-Wnt cells, also cloned from a MMTV-Wnt-1 tumor but with basal-like epithelial morphology and low expression of stem cell markers. Furthermore, CR promotes a mesenchymal-to-epithelial transition in the mammary gland by increasing the expression of the epithelial markers, such as E-cadherin, and decreasing the expression of mesenchymal markers, such as N-cadherin and fibronectin [115]. Taken together, these studies suggest an important role for the microenvironment in the response of stem cells (including cancer stem cells) to CR or CR mimetics targeting the mTOR pathway, and this will no doubt be an important and exciting research area in the coming years.

## Review

As summarized in Figure 1, this review considers lessons learned from CR and cancer research to discuss promising molecular targets for cancer prevention, particularly for breaking the link between obesity and cancer. Potential targets include components of energy-responsive growth factor and adipokine signaling pathways, inflammatory pathways, vascular regulators, autophagy regulators, and the sirtuin pathway. Clearly, no single pathway accounts for all of the anticancer effects of CR. As with most chronic disease intervention strategies, combination approaches involving lifestyle (including diet and physical activity) and pharmacological interventions that target multiple pathways (and that maximize efficacy and minimize adverse effects) are likely to be most successful for preventing cancer. Future studies aimed at further elucidating the mechanisms underlying the anticancer effects of CR, and that exploit this mechanistic information to target CR-responsive pathways will facilitate the translation of CR research into effective cancer prevention strategies in human beings.

## Conclusions

In this review we discussed possible mechanisms underlying the anticancer effects of CR, with emphasis on CR-associated changes in growth factor signaling, inflammation, and angiogenesis, as well as emerging evidence suggesting that autophagy and the sirtuin pathway may also play roles in the effects of CR on tumor development and progression. Several natural or synthetic agents have been shown to mimic some of the protective effects of CR and may thus represent new strategies for cancer prevention.

## Abbreviations

AMPK: AMP-activated kinase; CALERIE: Comprehensive Assessment of Long-Term Effects of Reducing Intake of Energy; COX: Cyclooxygenase; CR: Calorie restriction; DIO: Diet-induced obesity; GHR: Growth hormone receptor; IGF-1: Insulin-like growth factor-1; IGFBP: Insulin-like growth factor binding protein; IGF-IR: Insulin-like growth factor-1 receptor; IL: Interleukin; IR: Insulin receptor; JAK/STAT: Janus kinase and signal transducer activator of transcription; mTOR: Mammalian target of rapamycin; NF-κB: Nuclear factor κ-light-chain-enhancer of activated B-cells; TOR: Target of rapamycin; VEGF: Vascular endothelial growth factor.

## Competing interests

The authors declare that they have no competing interests.

## Authors’ contributions

All authors contributed sections to this review, and read and approved the final manuscript.
